# Biochemical and psychological markers of fatigue and recovery in mixed martial arts athletes during strength and conditioning training

**DOI:** 10.1038/s41598-025-09719-z

**Published:** 2025-07-07

**Authors:** Joanna Ostapiuk-Karolczuk, Hanna Dziewiecka, Patryk Bojsa, Mirosława Cieślicka, Monika Zawadka-Kunikowska, Kaźmierczak Wojciech, Anna Kasperska

**Affiliations:** 1 Department of Biological Sciences, Poznan University of Physical Education, Faculty of Sport Sciences in Gorzow Wielkopolski, Estkowskiego 13, Gorzów Wielkopolski, 66-400 Poland; 2 Department of Human Physiology, Nicolaus Copernicus University, Ludwik Rydygier Collegium Medicum in Bydgoszcz, Karłowicza 24, Bydgoszcz, 85-092 Poland

**Keywords:** MMA athletes, Hormones, Inflammation, Mood states, Fatigue, Biochemistry, Physiology

## Abstract

Mixed martial arts (MMA) training imposes significant physiological and psychological demands, increasing the risk of overtraining. This study aimed to assess temporal changes in biochemical and psychological fatigue and recovery markers during a structured 3-week strength and conditioning training program in professional MMA athletes. Ten male MMA athletes (26.2 ± 0.9 years; 7.0 ± 2.0 years of training experience) participated. Blood samples and psychological assessments (Profile of Mood States – POMS) were collected at four time points: pre-training (T-0), and after each training week (T-1, T-2, T-3). Analyzed biomarkers included cortisol, testosterone, catecholamines, hs-CRP, creatine kinase, and metabolic markers. Data were analyzed using repeated measures statistical tests to assess time-dependent changes. Cortisol and hs-CRP increased significantly after the first week (*p* < 0.01), while testosterone and catecholamines remained stable. Creatine kinase showed a persistent rise (*p* < 0.01), indicating muscle damage. Psychological assessments revealed increased fatigue, tension, and confusion, with reduced vigor (*p* < 0.05). Despite biochemical markers partially recovering, subjective fatigue persisted, highlighting a disconnect between physiological and psychological recovery. These findings highlight the importance of integrated monitoring strategies that include both biochemical and psychological assessments. The divergence between physiological and psychological recovery underscores the complexity of athlete fatigue and the need for integrated recovery interventions. Future research should explore long-term adaptations, sex-based differences, and targeted recovery strategies to optimize training, performance, and well-being in MMA athletes.

## Introduction

Preparing MMA athletes requires a multifaceted approach, integrating strength, speed, anaerobic, and aerobic conditioning with technical skill development in striking, wrestling, and grappling^1^. Although physiological demands during MMA competition remain understudied, partly due to the absence of heart rate monitoring in elite bouts^2,3^, data from other combat sports indicate high cardiovascular strain. Heart rates in boxing, kickboxing and taekwondo often reach 83–100% of maximum, and post-match rates of ~ 176 bpm have been reported in amateur fighters^4^. MMA athletes also demonstrate high aerobic capacity, with VO₂max values exceeding 55 mL/kg/min^5,6^. While these findings suggest significant anaerobic and aerobic demands, more MMA-specific research is needed across different weight classes and performance levels. The intensity and diversity of training impose significant physiological and psychological stress, further compounded by the challenges of balancing strength and endurance development, weight management, and maintaining effective regeneration profiles^7^.

Despite the demanding nature of MMA, studies investigating the combined physiological and psychological responses to training are scarce. Research in combat sports has traditionally focused on isolated physiological markers, such as cortisol, testosterone, creatine kinase, or inflammatory cytokines, without concurrent assessment of psychological stress^8^. This separation is often due to methodological limitations, including cross-sectional designs, single time-point sampling, or the absence of validated psychological tools. While some recent studies have attempted to explore both physiological and psychological responses, they have typically been conducted in other combat sports (e.g., judo, wrestling) and lacked longitudinal designs or sport-specific relevance to MMA athletes^9,10^. Yet, training-induced fatigue is a multidimensional phenomenon, shaped not only by biological stress responses but also by subjective factors such as mood fluctuations, perceived exertion, and motivational states. Therefore, a more integrated approach is needed to understand the complex recovery profiles and overtraining risks in MMA athletes^2,10,11^.

Biochemical markers such as testosterone, cortisol, creatine kinase, and catecholamines provide valuable insights into training stress and recovery. Testosterone, as a primary anabolic hormone, reflects an athlete’s adaptive and regenerative capacity. A decline in testosterone levels or the testosterone-to-cortisol (T/C) ratio, a well-established index of overtraining, may indicate an imbalance between anabolic and catabolic processes^12–14^. In contrast, cortisol is a catabolic hormone that regulates inflammation and mobilizes energy, but when chronically elevated, it may impair recovery and promote muscle breakdown^8^. Creatine kinase (CK) is commonly used to assess muscle damage and recovery status, with persistently high levels suggesting insufficient repair between training sessions^15^. Catecholamines, including adrenaline and noradrenaline, mediate acute cardiovascular and metabolic responses to exercise. While essential for immediate performance, excessive or blunted catecholamine responses can signal autonomic imbalance and inadequate adaptation to prolonged training^16^.

While hormonal markers offer physiological insights, they do not always align with subjective fatigue assessments. The Profile of Mood States (POMS) is widely used to evaluate mood disturbances and training adaptation^10^. High training loads, competition stress, and personal factors can dysregulate the hypothalamic-pituitary-adrenal (HPA) axis, leading to prolonged cortisol elevation and inflammatory imbalances^17^. Importantly, psychological indicators, such as increased perceived fatigue or reduced vigor, may precede measurable biochemical changes and can therefore function as early warning signs of overtraining. Chronic inflammation indicated by elevated hs-CRP and TNF-α has been associated with fatigue, impaired recovery, and mood disturbances^18,19^.

Although previous studies have investigated hormonal and inflammatory markers in combat sports, few have explored their dynamic relationship with subjective fatigue over a defined training period. Research on sports like judo and wrestling suggests that prolonged high-intensity training can elevate cortisol, trigger inflammation, and increase mood disturbances^9,12^. However, MMA-specific data remains rare, particularly on how training-induced stress affects physiological and psychological responses over time. In contrast to previous studies, this study introduces a comprehensive monitoring approach by incorporating weekly hormonal, inflammatory, and psychological assessments. This integrative design enables a more detailed understanding of fatigue, recovery patterns, and potential indicators of overtraining in elite MMA athletes.

This study aimed to assess the effects of a three-week MMA training period on cortisol, testosterone, catecholamines (adrenaline, noradrenaline), hs-CRP, and TNF-α,. It also examined changes in total protein, albumin, uric acid, urea, and white blood cell counts, alongside psychological responses measured by POMS. By integrating biological and psychological markers, the study sought to identify adaptation mechanisms, fatigue patterns, and potential overtraining indicators, contributing to optimized recovery and performance strategies in MMA athletes.

## Materials and methods

### General design

**Fig. 1:**
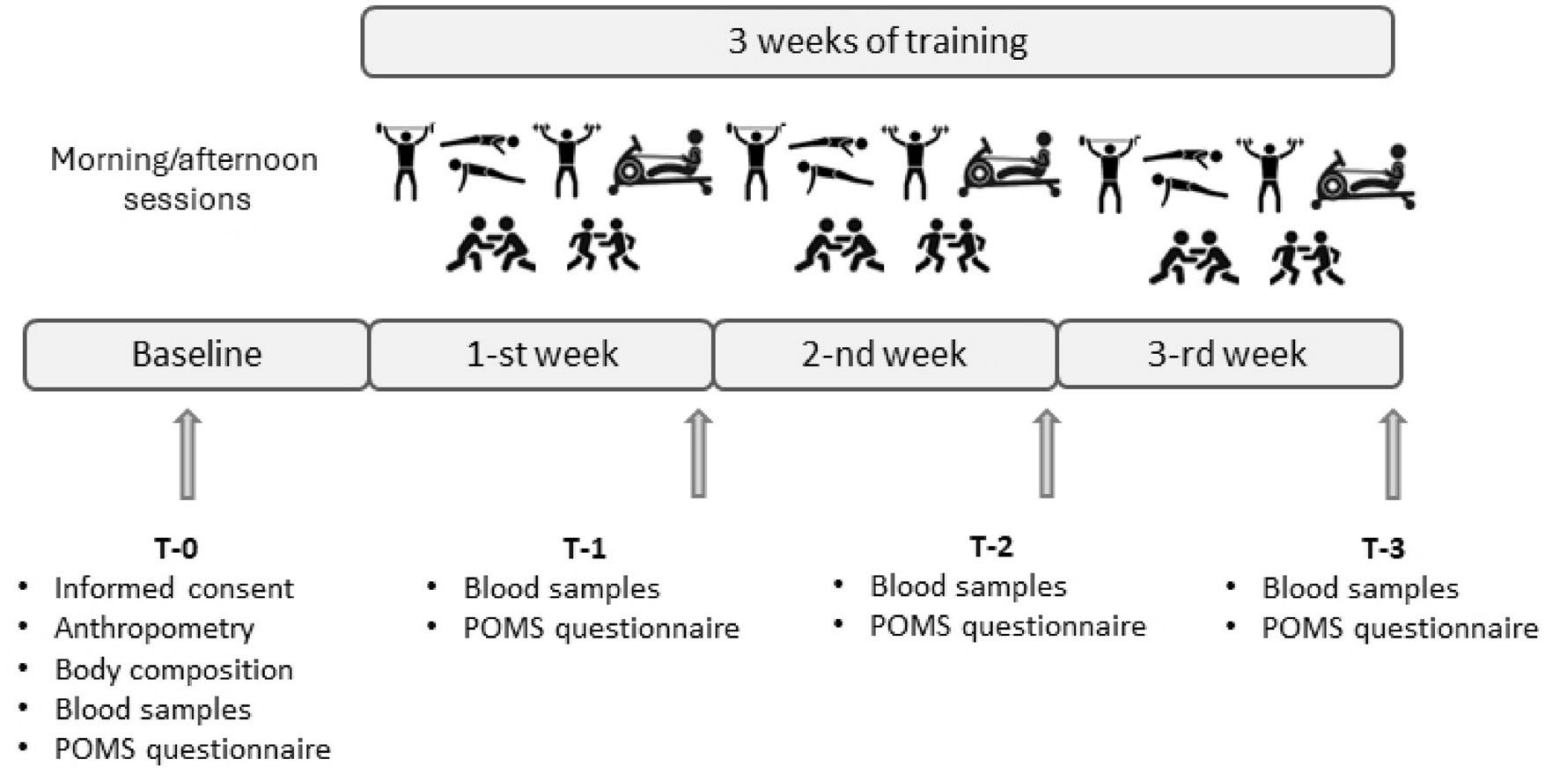
Study design.

The present study was conducted during a high-intensity conditioning program for combat athletes. Over three weeks, the training regimen focused on enhancing athletes’ strength and endurance. To reduce the influence of prior training, athletes were instructed to avoid vigorous exercise and resistance training for 48 h before baseline measurements. On the day before training began (T-0), all baseline measurements were taken, including anthropometric assessments, blood sample collection, and completion of questionnaires (Profile of Mood State – POMS). Then, blood samples were taken and questionnaires completed at the end of each training week: T-1 after the first week of training, and T-2 and T-3 after the subsequent weeks, respectively (see Fig. 1).

### Participants

The study was a nonrandomized observational time-series design, with measurements conducted at baseline and at the end of each training week. Sample size estimation was performed using G*Power (version 3.1.9.6; Heinrich Heine University, Dusseldorf, Germany) for repeated measures ANOVA with four levels of the within-subjects factor (T-0, T-1, T-2, T-3). Parameters were set as follows: an expected effect size of *f* = 0.4 an alpha level of 0.05, statistical power of 0.80, and a correlation among repeated measures of *r* = 0.50. The calculation determined that a minimum of 10 participants was required. A total of 10 participants were recruited to ensure the study’s validity despite potential dropouts. Participants were required to meet the following inclusion criteria: a minimum of five years of MMA training experience, with training at least three times per week, and at least two years of competitive fighting experience. They also had to be non-smokers, maintain a balanced diet, and refrain from using anabolic steroids, nutritional supplements, or other medications that could influence the study results. Participants were excluded from the study if they met any of the following criteria: any injury or clinical condition that prevented participation, use of anti-inflammatory drugs, or undergoing the weight-cutting period.

In total, 10 athletes (age 26,2 ± 0,9 years; height 179,4 ± 4,7 cm; body mass 77,5 ± 11,67 kg; % fat 12,3 ± 3,5; FFM (fat-free mass) 67,5 ± 8,7; training experience 7,0 ± 2,0 years) were recruited through convenience sampling from local MMA sports clubs based on voluntary participation and fulfillment of all inclusion and exclusion criteria. The small sample size is primarily due to the restrictive inclusion and exclusion criteria, which ensured a relatively homogenous and advanced group of athletes with comparable training experience. Additionally, all participants were required to undergo the same standardized training regimen, further narrowing the eligible pool to maintain consistency in the study’s methodology. All participants had been following comparable MMA training routines for at least two years before the study (minimum three sessions per week), ensuring baseline similarity. Additionally, to minimize variability, they were instructed to avoid intense training for 48 h before the baseline assessment. The body mass and composition were estimated at T-0 (baseline) using a bioelectrical impedance (BIA) by using Tanita Body Composition Analyzer BC-418 (Tokyo, Japan).

The duration of the training program was set at three weeks to capture the early physiological and psychological responses to intensified training. Previous studies in combat sports and high-intensity conditioning have demonstrated that significant alterations in hormonal markers (e.g., cortisol, testosterone), muscle damage indicators (e.g., creatine kinase), inflammatory markers (e.g., hs-CRP), and mood states (e.g., fatigue, vigor) can emerge within 1–3 weeks of intensive training stimuli. This timeframe was therefore considered sufficient to observe meaningful short-term adaptations and early signs of overreaching or fatigue accumulation, without extending into full adaptation or recovery cycles that might confound interpretation^9,12,17–19^.

To enhance internal validity and minimize inter-individual variability in training response, we deliberately recruited a homogeneous group of elite-level athletes. All participants had a minimum of five years of MMA training experience and at least two years of competitive fighting. This selection ensured a comparable baseline in training history, physiological conditioning, and psychological readiness. By standardizing participant characteristics and exposure to a uniform training protocol, we aimed to reduce confounding effects related to differential training backgrounds, thereby increasing the likelihood that observed changes could be attributed to the intervention itself.

### Ethical statement

 Athletes gave their free informed consent of participation in the study, which was conducted in accordance with the principles of the Declaration of Helsinki and approved by the Bioethical Committee at the Poznan University of Medical Science, Poland (Decision No. 546/11). This study had been registered on ClinicalTrials.gov ID: NCT0670599 (retrospectively registered, on 27.11.2024).

### Training protocol

Regular training was conducted 6 days per week with one rest day, consisting of two daily sessions, totaling approximately 18 h per week (3 h of training per day), excluding warm-ups and stretching routine (Table 1). Weight training was performed at an intensity of 75–85% of one-repetition maximum (1RM), focusing on compound movements. Circuit training followed a high-intensity interval format, using a 1:1 work-to-rest ratio (e.g., 30 s of maximal effort followed by 30 s of rest), targeting both aerobic and anaerobic capacity. Where applicable, aerobic efforts were estimated to occur at 80–90% of VO₂max based on prior athlete results. Evening sparring sessions were performed at high intensities (RPE 7–9), simulating fight conditions, while technical drills and morning sessions (RPE 3–6) were lower in intensity and focused on skill refinement. Session intensities were monitored using the modified Borg Rating of Perceived Exertion (RPE) scale. During the entire observation period, training loads remained constant. Training sessions started with a 10-minute warm-up with low-intensity exercises, and each session was finished with a 10-minute cooldown.

**Table 1:** Weekly training schedule for MMA athletes.

Day	Session 1 (8:00 AM–8:30 AM)	RPE	Session 2 (6:00 PM–7:30 PM)	RPE
Monday	Circuit training	5–8	Technique/drills	3–6
Tuesday	Weight training	5–6	Sparring/wrestling	7–9
Wednesday	Technique/drills	3–6	Sparring/wrestling	6–7
Thursday	Circuit training	5–8	Weight training	5–6
Friday	Technique/drills	3–6	Sparring/wrestling	7–9
Saturday	Weight training	5–6	Circuit training	5–8
Sunday	Rest day	–	Rest day	–

Training consists of circuit training, weight training, sparrings, and technique/drills. Circuit training focused on improving muscular endurance. During each station, participants performed exercises for 30 s, with 30 s of rest. Stations included bodyweight squats, push-ups, jumping lunges, plank with shoulder taps, medicine ball slams, rowing ergometers, box jumps or step-ups, battle rope waves, kettlebell swings, and burpees. Weight training exercises included deadlifts, barbell back squats, box jumps, bench presses (barbells or dumbbells), weighted push-ups, and pull-ups. Techniques/drills included striking techniques and drills, grappling, and ground techniques. Sparring/wrestling rounds of open-skill sparring or wrestling. Standard training sessions were about 10 rounds of 5 min each with 2–3 min break between. Sparring aimed to put learned skills into practice. The training schedule was designed to ensure a balanced focus on endurance, strength, technique, and live practice while maintaining a consistent training load throughout the observation period.

The perceived intensity of each training category and the overall session was assessed by asking each participant to individually record their RPE (rate of perceived exertion) using the Foster^20^ sessional RPE 0–10 scale. Participants were instructed to provide their RPE 10–30 min after the completion of the entire training session to account for a holistic evaluation of perceived effort. To classify intensity levels, the following delineations were applied: low intensity (RPE ≤ 4), moderate intensity (RPE 5–6), and high intensity (RPE ≥ 7), as described in previous studies^2,21^

Although individual recovery strategies such as nutrition, hydration, and sleep were not formally standardized or monitored, all participants were instructed to maintain their regular routines throughout the study period to reduce variability in recovery-related factors.

### Mood state evaluation (POMS)

The POMS questionnaire^22,23^ was utilized to assess mood during the training at T-0, T-1, T-2, and T-3. The original version comprises six scales: anger, fatigue, confusion, depression, tension, and vigor. Respondents rate the intensity of their mood state over the past week, described by each adjective, on a scale ranging from 0 (definitely not) to 4 (definitely yes). All questionnaires were completed in the morning, immediately after blood sample collection and, before the start of the training session, to minimize the influence of diurnal variation and acute exercise effects on mood state.

### Laboratory tests

Blood samples were drawn at T-0, T-1, T-2, and T-3 under standardized conditions (morning, fasting, post-rest). All blood samples were collected by the same experienced nurse. Hemoglobin, hematocrit, and cell counts were measured immediately (SYMEX K-4500, Poland). Serum was separated by centrifugation at 3,000 rpm for 10 min at 4 °C and stored at − 80 °C until further analysis.

#### Biochemical determinations

The concentration of catecholamines: adrenaline and noradrenaline in serum was measured using the 2-CAT (A-N) ELISA Fast-Track kit (LDN, Germany). The intra-assay coefficient of variation (%CV) was 9.3% and 11.7% for adrenaline and noradrenaline, respectively. The responsiveness of the adrenal medulla to sympathetic nervous activity was assessed using the calculated adrenaline-to-noradrenaline ratio. The concentration of cortisol, testosterone, and hs-CRP in serum was measured using the immunodiagnostic assays: DRG Cortisol ELISA, DRG Testosterone ELISA DRG hs-CRP. The intra-assay precision (%CV) was 11%, 4,6%, 4,4%, and 5,4% for cortisol, testosterone, and hs-CRP, respectively. The testosterone/cortisol ratio was calculated to estimate the responsiveness of the hypothalamic-pituitary-adrenal axis to training stress. Total creatine kinase (CK) activity was measured using a spectrophotometric method with a reagent kit (BioMaxima S.A., Poland; detection limit: 7 U/L).

The levels of total protein (TP), albumin (A), uric acid (UA), and urea (U) were determined using ready-made biochemical reagents kits (BioMaxima, Lublin, Poland), according to the manufacturer’s protocol and analysis procedures were validated with the use of multiparametric control serum (BIOLABO S.A.S, Maizy, France). All biochemical parameters were determined using the microplate/cuvette absorbance reader (Spectrostar Nano, BMG Labtech).

#### Statistical analyses

 The statistical analyses were carried out with the STATISTICA v. 13.0 software package (Stat–Soft, Kraków, Poland). Descriptive statistics, including mean and SD, were used to visualize immediate trends and patterns. All variables used in the study were checked for normality of distribution before the analyses (Shapiro-Wilk test). Levene’s test was used to verify the homogeneity of variance. To assess differences across the four time points (T-0, T-1, T-2, and T-3), one-way repeated-measures ANOVA was applied. When significant main effects were observed, pairwise comparisons between time points were conducted using paired-sample t-tests with Bonferroni correction, which appropriately controls for multiple comparisons in within-subject designs. Cohen`s d values were calculated by the measured variables to quantify the effect size. Using Cohen`s criteria, the effect size was interpreted as small (0.2), moderate (0.5), and large (0.8)^24^. Pearson correlation coefficients were used to examine relationships between biochemical and psychological markers. For each correlation, the *r* value, *p*-value, and 95% confidence interval (CI) were reported. The strength of correlations was interpreted according to the scale proposed by Hopkins^25^: trivial (*r* < 0.1), small (0.1 ≤ *r* < 0.3), moderate (0.3 ≤ *r* < 0.5), large (0.5 ≤ *r* < 0.7), very large (0.7 ≤ *r* < 0.9), nearly perfect (0.9 ≤ *r* < 1.0), and perfect (*r* = 1.0). A significance threshold of *p* ≤ 0.05 was applied. There were no missing data recorded during the study.

## Results

**Fig. 2:**
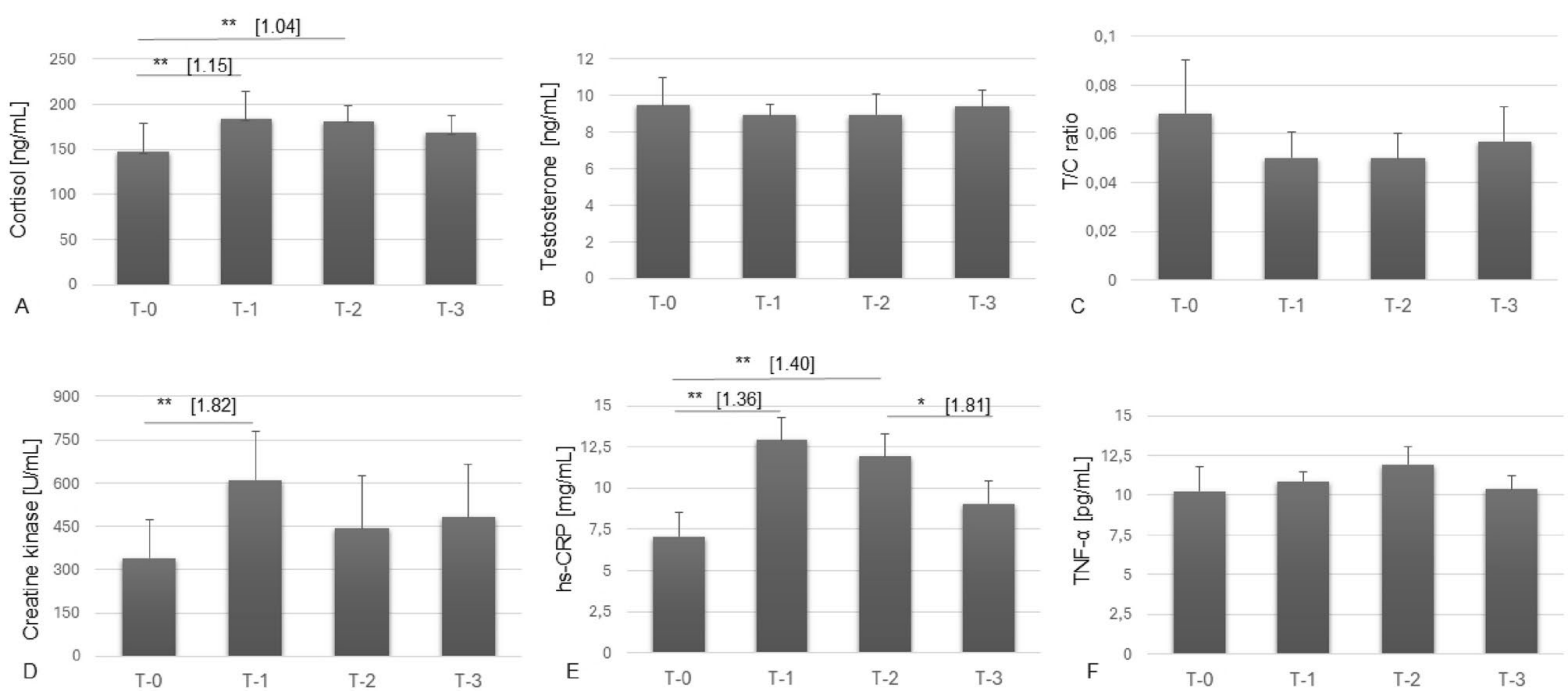
Changes in cortisol, testosterone, T/C ratio, creatine kinase, hs-CRP, and TNF-α in MMA athletes during a three-week training program. Significant differences between time points are indicated: *p < 0.05, **p < 0.01. Values are presented as means ± standard deviation (SD). Cohen’s d values ​​are given in square brackets.

Cortisol concentrations significantly increased after the first week of training (T-0 vs. T-1, *p* < 0.01, *d* = 1.15), followed by stabilization at subsequent time points (T-0 vs. T-2, *p* < 0.05, *d* = 1.04). Despite a slight reduction at T-3, cortisol levels remained elevated compared to baseline, with a large effect size observed between T-0 and T-3 (*d* = 1.04), indicating a meaningful physiological change (see Fig. 2A). Testosterone concentrations remained unchanged throughout the observation period, with no significant differences between time points. The largest effect size was observed between T-0 and T-1 (*d* = 0.88) (Fig. 2B). The testosterone-to-cortisol (T/C) ratio exhibited a slight, non-significant decrease after the first week of training and remained stable thereafter. Notably, a meaningful effect was observed between T-0 and T-1 (*d* = 1.33), despite the absence of statistical significance (Fig. 2C).

Creatine kinase levels significantly increased following the first week of training (T-0 vs. T-1, *p* < 0.01, *d* = 1.82). Although a downward trend was observed at T-2 and T-3, these reductions were not statistically significant (Fig. 2D). hs-CRP concentrations increased significantly from T-0 to T-1 (*p* < 0.001, *d* = 1.36) and T-0 to T-2 (*p* < 0.01, *d* = 1.40), followed by a significant reduction at T-3 compared to T-2 (*p* < 0.05, *d* = 1.81) (Fig. 2E). TNF-α levels did not change significantly over the study period, with the largest effect size observed between T-0 and T-2 (*d* = 0.83) (Fig. 2F).

Adrenaline levels remained relatively stable throughout the training period, with only minor fluctuations across time points. The largest effect size was observed between T-1 and T-3 (*d* = 0.21), however, this is still considered a small effect (see Table 2). Similarly, noradrenaline levels exhibited minimal variability, with small effect sizes for all pairwise comparisons, and the largest effect size observed between T-0 and T-3 (*d* = 0.21), which, although the highest among comparisons, is still classified as a small effect (Table 2). The adrenaline-to-noradrenaline (A/NA) ratio remained consistent across time points, with the highest observed effect size occurring between T-0 and T-3 (*d* = 0.07), although this still represents a small effect (Table 2).

**Table 2:** Changes in adrenaline, noradrenaline, A/NA ratio, urea, uric acid, albumin, and total protein selected biochemical markers in MMA athletes during a three-week training program.

	T-0	T-1	T-2	T-3	d-Cohen					
					T-0 vs. T-1	T-0 vs. T-2	T-0 vs. T-3	T-1 vs. T-2	T-1 vs. T-3	T-2 vs. T-3
Adrenaline (pg/mL)	151.55 ± 9.82	148.99 ± 41.07	149.9 ± 41.76	156.57 ± 30.41	0.06	0.04	0.14	0.02	0.21	0.18
Noradrenaline (pg/mL)	781.71 ± 71.49	760.47 ± 37.55	765.13 ± 96.34	767.18 ± 65.64	0.39	0.20	0.21	0.7	0.13	0.03
A/NA ratio	0.19 ± 0.5	0.19 ± 0.05	0.19 ± 0.05	0.21 ± 0.05	0.00	0.00	0.07	0.00	0.04	0.04
Urea (mg/dL)	72.02 ± 15.20	78.50 ± 16.40	97. 13 ± 16.35	75.11 ± 9.82	0.12	1.59	0.25	1.42	0.26	1.68
Uric acid (mg/dL)	6.51 ± 0.98	7.60 ± 3.25	8.62 ± 2.85^b^	7.13 ± 1.62	0.52	1.10	0.48	0.33	0.19	0.67
Albumin (mg/dL)	4.63 ± 0.35	4.42 ± 0.30	4.50 ± 0.36	5.02 ± 0.33^e^	0.60	0.37	1.15	0.24	1.90	1.51
Total protein (g/dL)	6.39 ± 0.75	6.01 ± 0.54	7.00 ± 1.17	7.80 ± 2.45^c, e.f^	0.59	0.64	8.88	1.66	1.20	0.44

Results are presented as means ± standard deviation. Effect sizes (d-Cohen) for pairwise comparisons between time points are included. Significant differences are denoted as follows: ^b^significant difference compared to T-0; ^e^significant difference compared to T-1; ^f^significant difference compared to T-2. The significance level was set at *p* < 0.05.

Urea concentrations showed a marked but non-significant increase from baseline (T-0) to T-1. By T-3, urea levels declined, though they remained above baseline. Uric acid levels followed a similar trend, increasing marginally significantly from T-0 to T-2 (*p* = 0.05288, *d* = 1.10). Levels at T-3 decreased slightly but did not return to baseline (see Table 2). Albumin concentrations slightly decreased from T-0 to T-1, stabilized at T-2, and significantly increased by T-3 (T-1 vs. T-3, *p* < 0.01, *d* = 1.90). Total protein levels exhibited a consistent upward trend, peaking at T-3 (T-0 vs. T-3, *p* < 0.01, *d* = 8.88) (Table 2).

Leukocyte concentrations increased significantly from T-0 to T-2 (*p* < 0.05, *d* = 1.69) before stabilizing at T-3, with a nonsignificant slight decrease from T-2 (*d* = 0.13) (see Table 3). Neutrophil counts followed a similar trend, though changes were not significant, with the largest effect size observed between T-0 and T-3 (*d* = 2.05) (Table 3). Lymphocyte concentrations remained relatively stable across all time points, showing a slight upward trend, with the largest effect size observed between T-0 and T-3 (*d* = 0.64), though overall changes were minor (Table 3). Monocyte levels gradually decreased, with a notable difference between T-0 and T-3 (*d* = 0.92) and the largest decrease between T-1 and T-3 (*d* = 0.78) (Table 3). Eosinophil counts showed minimal variation, with a slight increase from T-0 to T-1 (*d* = 0.10) but no significant changes thereafter. Basophil levels fluctuated slightly, with a slight increase from T-0 to T-2 (*d* = 0.25) followed by a decrease at T-3 (T-2 vs. T-3, *d* = 0.60). The largest difference was observed between T-0 and T-3 (*d* = 0.33) (Table 3).

**Table 3:** Changes white blood cell count in MMA athletes over a 3-week training program.

	T-0	T-1	T-2	T-3	d-Cohen					
					T-0 vs. T-1	T-0 vs. T-2	T-0 vs. T-3	T-1 vs. T-2	T-1 vs. T-3	T-2 vs. T-3
Leukocyte (10^9^/L)	6.88 ± 1.15	9.13 ± 1.72	9.26 ± 1.66^a^	9.04 ± 1.73	1.57	1.69	1.50	0.88	0.05	0.13
Neutrophil (10^9^/L)	3.12 ± 1.16	4.76 ± 0.92	4.87 ± 1.14	4.79 ± 0.47	1.58	1.52	2.05	0.11	0.04	0.10
Lymphocyte (10^9^/L)	3.09 ± 0.78	3.14 ± 0.68	3.29 ± 0.66	3.59 ± 0.78	0.07	0.27	0.64	0.22	0.62	0.42
Monocyte (10^9^/L)	0.77 ± 0.23	0.73 ± 0.20	0.67 ± 0.19	0.59 ± 0.16	0.19	0.48	0.92	0.31	0.78	0.46
Eosinophil (10^9^/L)	0.28 ± 0.20	0.30 ± 0.20	0.26 ± 0.17	0.26 ± 0.22	0.10	0.11	0.10	0.22	0.19	0.00
Basophil (10^9^/L)	0.10 ± 0.05	0.10 ± 0.05	0.11 ± 0.03	0.08 ± 0.07	0.00	0.25	0.33	0.25	0.33	0.60

Results are presented as means ± standard deviation. Effect sizes (d-Cohen) for pairwise comparisons between time points are included. Significant differences are denoted as follows: a – significant difference compared to T-0. The significance level was set at *p* < 0.05.

**Fig. 3:**
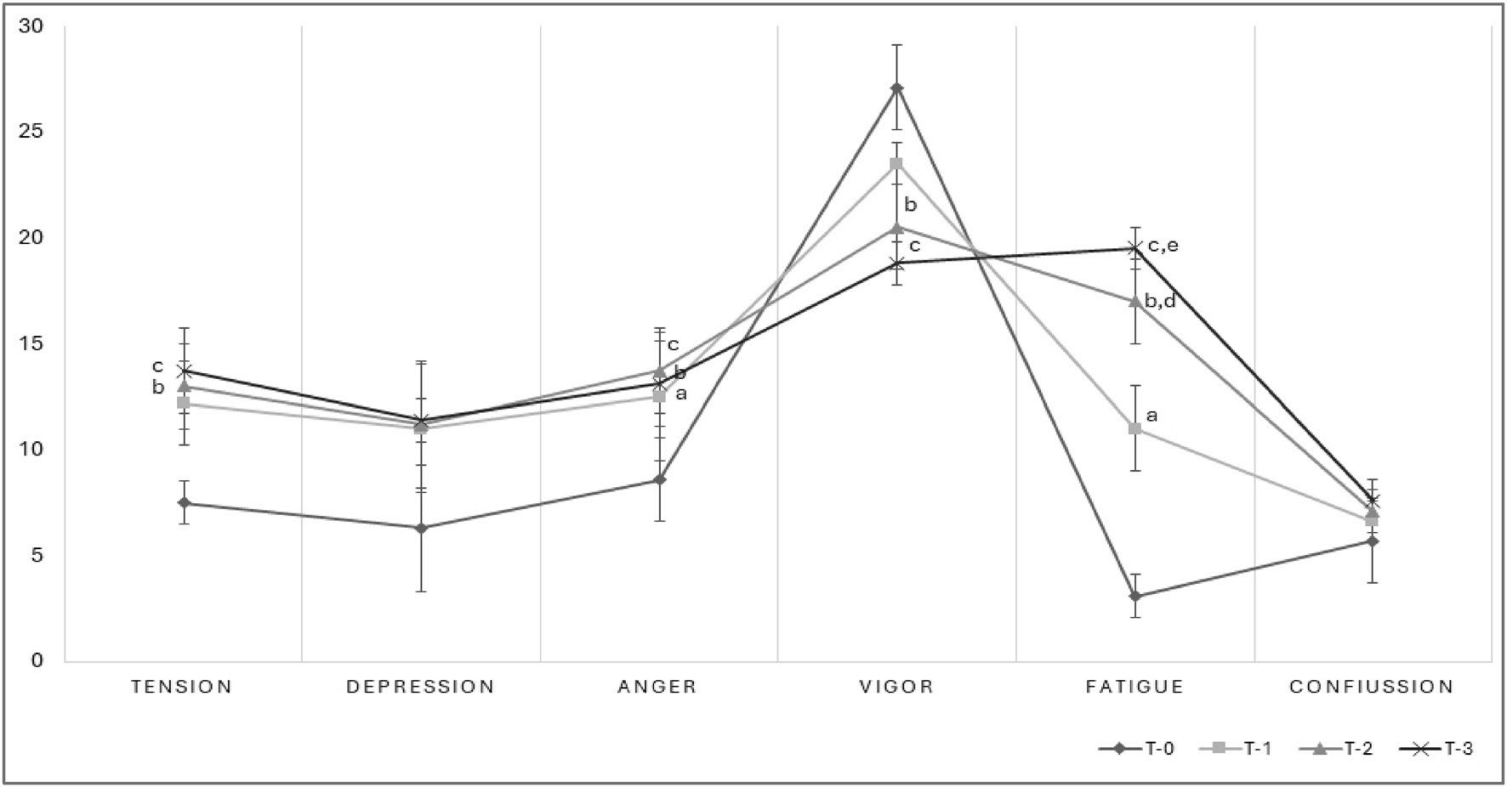
Changes in Profile of Mood States (POMS) scores in MMA athletes over a 3-week training program. Results are presented as means ± standard deviation. Significant differences are denoted as follows: a,b – significant difference compared to T-0; d, e - significant difference compared to T-1. The significance level was set at *p* < 0.05.

The POMS profile of each time-point is shown in Fig. 3. Tension scores showed a significant increase from T-0 to T-1 (*p* < 0.0001; *d* = 1.42), with a steady rise continuing through T-2 (T-0 vs. T-1 *p* < 0.0001; *d* = 1.62) and T-3 (T-0 vs. T-3 *p* < 0.0001; *d* = 1.75). The highest tension scores were observed at T-3, indicating a progressive trend throughout the training period. Depression scores remained relatively stable across all time points, with no significant differences observed between T-0, T-1, T-2, and T-3. Anger scores increased notably from T-0 to T-1 (T-0 vs. T-1 *p* < 0.01; *d* = 0.88) and continued to rise through T-2 (T-0 vs. T-2 *p* < 0.001, *d* = 1.52) and T-3 (T-0 vs. T-3 *p* < 0.01; *d* = 1.24). By T-3, anger levels stabilized, showing no further significant changes from T-2. Vigor scores exhibited a significant decrease from T-0 to T-1 (*p* < 0.05; *d* = 1.44). This downward trend continued across T-2 (T-0 vs. T-2 *p* < 0.0001; *d* = 1.81) and T-3 (*p* < 0.0001; *d* = 2.59), with the lowest vigor levels recorded at T-3, indicating a substantial reduction in positive mood states over time. Fatigue scores increased significantly from T-0 to T-1 (*p* < 0.0001; *d* = 2.59) and continued to rise at T-2 (T-0 vs. T-2 *p* < 0.0001; *d* = 4.48) and T-3 (T-0 vs. T-3 *p* < 0.0001; *d* = 6.56). The highest fatigue scores were observed at T-3, showing a progressive accumulation of fatigue during the training period. Confusion scores rose slightly from T-0 to T-1 and continued to increase at T-2 and T-3, with the highest scores recorded at T-3. However, the changes in confusion levels were less pronounced compared to other mood states (Fig. 3).

There were a moderate negative correlations observed between cortisol and testosterone (*r* = -0.47, 95% CI: (− 0.70, − 0.0.18), *p* < 0.01), and a small negative correlation between cortisol and vigor (*r* = − 0.37, 95% CI: (− 0.62, − 0.02), *p* < 0.05). A small positive correlation was found between cortisol and tension (*r* = 0.35, 95% CI: (0.02, 0.61), *p* < 0.05). Testosterone showed a moderate negative correlation with tension (*r* = − 0.36, 95% CI: (− 0.62, − 0.04), *p* < 0.05), a strong negative correlation with fatigue (*r* = − 0.66, 95% CI: (0.53, 0.78) *p* < 0.001), and a small positive correlation with vigor (*r* = 0.32, 95% CI: (− 0.04. 0.58), *p* < 0.05). Creatine kinase moderately positively correlated with confusion (*r* = 0.35, 95% CI: (0.03, 0.62), *p* < 0.05) and with fatigue (*r* = 0.32, 95% CI: (− 0.01, 0.60), *p* < 0.05). Also, among POMS scores, vigor was strongly negatively correlated with tension (*r* = − 0.68, 95% CI: (− 0.80, − 0.52), *p* < 0.001) and fatigue (*r* = − 0.79, 95% CI: (− 0.88, − 0.65), *p* < 0.001).

## Discussion

As highlighted in the introduction, MMA training integrates strength, endurance, and technical skills, resulting in multifaceted physiological demands and increasing fatigue. Although biochemical markers of fatigue, such as cortisol and inflammation markers (hs-CRP), in our study showed a clear decrease in the final phase of the observed period, suggesting a partial adaptive response to training loads, subjective assessments using the Profile of Mood States (POMS) indicate that athletes continue to experience pronounced fatigue and a significant decline in vigor. This divergence between physiological and subjective responses underscores the complexity of training adaptation, where biochemical recovery does not necessarily equate to psychological well-being.

According to Broodryk et al.^26^, cortisol plays a role not only in the physiological response to exercise but also in psychological processes, indicating a connection between the heightened activity of the central nervous system and the neuroendocrine system, along with the subjective perception of training. The initial increase in cortisol during the first two weeks (T-1 and T-2) followed by a decline in the final week (T-3) suggests an acute stress response with partial adaptation. This trend aligns with previous findings in MMA athletes, where high-intensity training initially elevated cortisol levels, followed by a return to baseline as adaptation occurred^27^. A similar pattern was observed in a 12-week MMA athletes’ training program, where cortisol increased during the preparation phase before normalizing during the recovery phase^8^. Although cortisol levels increased significantly during the first week of training, the concentrations observed in our study remained below thresholds typically associated with clinical stress-related maladaptation, which are generally considered to exceed 700–800 nmol/L in serum^28,29^. This suggests that while the hormonal response was pronounced, it may reflect a functional adaptation to training load rather than pathological dysregulation.

During our study testosterone levels remained stable, leading to a non-significant decrease in the testosterone-to-cortisol (T/C) ratio. This hormonal imbalance is often associated with training-induced fatigue and impaired recovery, as cortisol promotes catabolic activity, while testosterone supports muscle repair and anabolic processes^30^. The absence of a compensatory testosterone response suggests that prolonged high-intensity training may limit anabolic recovery, reinforcing the importance of tailored resistance training, nutrition, and recovery strategies^31,32^. Although acute resistance training often stimulates anabolic hormone responses, we observed no significant changes in testosterone concentrations. This could be due to the relatively short duration of the intervention, which may not have been sufficient to elicit long-term endocrine adaptations. Additionally, given the participants’ high training experience (average of seven years), it is likely that their hormonal systems were already adapted to intensive training, resulting in a more stable testosterone response despite physiological stress^30,33^.

Cortisol’s influence extends beyond physiological stress, as elevated levels have been linked to increased tension, fatigue, anxiety, and depression while reducing vigor^34^. In our study, cortisol levels correlated negatively with vigor (*r* = -0.37) and positively with tension (*r* = 0.35), reinforcing its role in psychological stress regulation. These findings align with research in other combat sports, where prolonged training elevated cortisol levels and led to increased mood disturbances^9^.

POMS data revealed that fatigue and tension scores increased progressively throughout the study, despite the observed decline in cortisol during the final week. This suggests that subjective fatigue behind physiological adaptation emphasizes the need for integrated monitoring that includes both biochemical and psychological assessments^35^. A similar trend was noted in elite judoists undergoing competitive training, where rising cortisol levels were linked to increased psychological strain and reduced vigor^9^.

Testosterone levels, on the other hand, showed a positive correlation with vigor (*r* = 0.32) and a negative correlation with tension (*r* = -0.36) and fatigue (*r* = -0.66), indicating their role in maintaining mood stability and motivation. Similar findings in combat sports suggest that higher testosterone levels contribute to psychological readiness and resilience^32^. Salvador et al.^36^ also reported that testosterone fluctuations influenced mood states in judo athletes, with higher levels associated with increased confidence and motivation. The stable testosterone levels in this study may have helped buffer some of the psychological strain despite the ongoing physiological stress.

Adrenaline and noradrenaline levels remained stable throughout the three-week training period, with only minor fluctuations. The consistent adrenaline-to-noradrenaline (A/NA) ratio suggests that training loads were within the athletes’ adaptive capacity, avoiding excessive sympathetic activation. This aligns with findings that structured, well-regulated training can lead to a blunted catecholamine response at submaximal intensities, improving autonomic efficiency^16^. In contrast, excessive or prolonged training often results in heightened catecholamine activity due to chronic stress or, conversely, blunted responses indicative of overtraining^15^. Research on combat athletes suggests that acute high-intensity sessions lead to transient spikes in catecholamine levels, followed by adaptation over time^37^. The lack of significant fluctuations in this study suggests that while training was demanding, it did not exceed the threshold required to trigger excessive sympathetic exhaustion.

Muscle damage was evident in the study, as CK levels significantly increased after the first training week (T-1), suggesting substantial muscular stress. Although CK levels declined slightly in subsequent weeks (T-2 and T-3), they remained elevated, indicating persistent muscle damage. This trend has been observed in MMA athletes, where prolonged high-intensity training leads to cumulative muscle breakdown when recovery is insufficient^8,32^. Notably, several participants exceeded 1,000 U/L—a threshold commonly referenced in sports medicine as indicative of substantial skeletal muscle damage, particularly following eccentric or high-volume strength training^38,39^. 

These elevations were greater than those typically reported in elite boxers and karate athletes under standard training conditions^39,43^. In combat sports like MMA, the high mechanical load and repeated impacts likely contribute to such elevations in muscle damage markers^44^. To sum up, the MMA athletes’ hormonal and muscle-damage responses were more pronounced than those reported for comparable training bouts in boxing or karate, and comparable to or higher than the peaks documented in high-intensity judo or wrestling , underlining the heavy physiological strain of MMA competition.

Furthermore, uric acid levels remained elevated throughout the study, reflecting increased metabolic stress and purine catabolism. This suggests a heightened demand for ATP turnover and oxidative stress management, which is consistent with findings in MMA athletes undergoing intense training^45^. Similar trends have been reported in combat sports, where uric acid levels rose during periods of anaerobic exertion, emphasizing the metabolic strain of high-intensity exercise^8,46^.

In our study, POMS results reflected biochemical changes, with fatigue scores rising significantly from T-0 to T-3, while vigor declined. The positive correlation between CK and fatigue (*r* = 0.32) suggests that greater muscle damage contributes to higher perceived exertion and delayed recovery, reinforcing the link between physiological strain and psychological fatigue. Similar findings were reported by Berriel et al.^47^ in volleyball players.

Additionally, the increase in fatigue and decline in vigor mirrored the trend in uric acid levels. Elevated uric acid correlated positively with fatigue (*r* = 0.29) and negatively with vigor (*r* = -0.27), indicating a potential association with greater physiological stress and reduced psychological well-being, consistent with previous research^48^.

Inflammatory markers further confirmed this physiological strain, as hs-CRP levels significantly increased from baseline (T-0) to T-1, suggesting an acute inflammatory response to muscle damage. Although hs-CRP levels declined slightly in later weeks, they did not return to baseline, indicating prolonged low-grade inflammation. The peak values of hs-CRP observed in some athletes exceeded 3.0 mg/L, a threshold commonly used to indicate low-grade systemic inflammation^49^. While this does not imply clinical illness, it may reflect a physiologically relevant inflammatory response to repeated high-intensity training, which warrants monitoring during prolonged or intensified training blocks. In contrast, TNF-α levels remained stable throughout the training period, suggesting that systemic inflammation was controlled despite localized muscle stress. Similar findings have been reported in elite wrestlers, where hs-CRP increased after prolonged training, but TNF-α responses remained minimal^30^ and boxers^50^. These findings suggest that in well-regulated training regimens across combat sports, the increase in hs-CRP serves as a marker of acute-phase inflammation without triggering significant TNF-α-mediated systemic inflammation, reflecting the balance between tissue repair and immune homeostasis.

Furthermore, increases in leukocyte and neutrophil counts from T-0 to T-1, which remained elevated through T-2 and T-3, highlight an acute immune response to muscle damage and systemic inflammation. Elevated leukocyte and neutrophil levels are characteristic of the body’s immediate response to physical stress and inflammation, as these cells play a significant role in clearing debris and facilitating tissue repair. These findings align with previous studies on combat sports athletes, where intense training elicited similar immune and inflammatory responses^44^. Additionally, our findings revealed a correlation between uric acid and vigor, which may reflect the neuromodulatory role of uric acid. As an endogenous antioxidant, uric acid may influence mood regulation and central fatigue via purinergic and oxidative pathways^51^.

In our study, POMS results mirrored the inflammatory response, with increasing fatigue and tension and declining vigor, reaching the lowest point at T-3. Elevated hs-CRP levels alongside worsening mood suggest that systemic inflammation may contribute to both physical fatigue and psychological distress. This aligns with research linking heightened inflammatory markers to disrupted neurotransmitter signaling, increased fatigue, and reduced motivation^10,17^. Similar trends have been observed in athletes, where elevated hs-CRP and cytokines correlate with higher fatigue and lower vigor^8^. Findings in judo athletes further support this, showing increased inflammation and hormonal changes during competition phases, leading to fatigue and tension^9,43^. The persistence of low-grade inflammation and psychological fatigue underscores the need for effective recovery strategies to mitigate inflammation and support mental resilience in athletes. Although statistically significant, the biological–psychological correlations observed in our study were moderate in strength and likely reflect multifactorial interactions between systemic inflammation, neuromuscular stress, and central fatigue pathways.

POMS results indicate significant mood disturbances, including increased tension, fatigue, and confusion, alongside decreased vigor, reflecting the cumulative impact of physical and psychological stress. Similar effects have been reported in combat sports, where intense training and weight management contribute to psychological strain. In professional MMA athletes, a five-week fight camp led to mood disturbances, particularly during rapid weight loss^35^. In judo and Brazilian jiu-jitsu, elevated anger and tension were linked to improved performance, but excessive negative mood states impaired cognitive function, motivation, and recovery^52^. These findings highlight the importance of psychological support to maintain optimal mental resilience in athletes.

The interplay between hormonal responses, muscle damage, and mood states underscores the complexity of adaptation during mixed strength-endurance training. Monitoring these parameters alongside mood assessments provides valuable insights into an athlete’s readiness and recovery. Tailored interventions, including nutritional support, recovery protocols, and psychological strategies, are essential to optimize performance and prevent overtraining in MMA athletes. Despite stable testosterone levels in this study, elevated cortisol and inflammatory markers likely contributed to increased fatigue and reduced vigor, a pattern consistent with findings in other combat sports.

This study is limited by a small sample size, short duration, and lack of a control group, restricting generalizability and long-term assessment. Uncontrolled external factors such as nutrition, sleep, and psychological stress may have influenced results. The study did not assess direct performance outcomes, limiting its applicability to competition readiness. Addressing these limitations in future research will provide a more comprehensive understanding of training, recovery, and performance interactions. Although the sample size was statistically justified, the small number of participants may limit the generalizability of the findings and reduce the sensitivity to detect small effect sizes between time points. Sleep parameters (duration and quality) were not formally monitored, although participants were instructed to maintain consistent sleep routines throughout the training period. We acknowledge this as a potential limitation, as previous studies have shown that sleep disruption can influence inflammatory and muscle damage markers^53,54^. Additionally, no dietary intake was standardized or recorded, despite its known influence on inflammatory, hormonal, and psychological responses. Future studies should consider implementing structured dietary logs or standardized meals, as well as incorporating direct performance metrics, such as power output, strength, or reaction time, to improve control over confounding factors and enhance the practical relevance of biomarker- and mood-based monitoring in combat sports.

## Conclusions and practical implications

This study provides crucial insights into the physiological and psychological impact of high-intensity MMA training, highlighting the necessity of a multifaceted approach to athlete monitoring. The observed decline in biochemical stress markers, such as cortisol and creatine kinase, suggests partial physiological adaptation however, the persistent increase in subjective fatigue and decrease in vigor reveals a critical disconnect between biochemical recovery and psychological well-being. This discrepancy underscores the study’s significance in emphasizing the need for comprehensive monitoring strategies that integrate both physiological and psychological assessments.

By demonstrating that biochemical markers alone are insufficient indicators of overall recovery, this research reinforces the importance of incorporating mental health evaluations into athlete management. The findings suggest that neglecting psychological strain could increase the risk of chronic stress, burnout, and impaired performance, even in athletes who show recovery on the biochemical level. Therefore, implementing stress management strategies, cognitive-behavioral techniques, and mindfulness training should be considered essential components of training programs. In practical terms, regular psychological screening using tools like the Profile of Mood States (POMS) can help detect early signs of fatigue and mood disturbances. Recovery strategies such as mindfulness-based relaxation, guided breathing, or psychological support may assist in managing stress during intensive training periods. Sleep hygiene (e.g., consistent sleep–wake routines, reduced screen exposure) and antioxidant-rich nutrition can further aid recovery. Importantly, recovery protocols should be individualized based on hormonal and mood fluctuations. Periodized tapering adjusted to psychological and biological markers may also help reduce overreaching and enhance performance.

Furthermore, this study sets a foundation for future research on long-term training adaptations, particularly concerning hormonal, inflammatory, and psychological responses. Examining sex-specific differences and assessing targeted recovery interventions could provide deeper insights into optimizing training and recovery strategies for MMA athletes. The findings of this study serve as a critical step toward a more comprehensive understanding of athlete adaptation, ultimately contributing to improved performance, resilience, and overall well-being in high-intensity combat sports.

## Data Availability

Due to confidentiality restrictions, the dataset generated and analyzed in this study is not publicly available but can be accessed from the corresponding author upon reasonable request.
